# *Nitraria retusa* fruit prevents penconazole-induced kidney injury in adult rats through modulation of oxidative stress and histopathological changes

**DOI:** 10.1080/13880209.2016.1278455

**Published:** 2017-02-15

**Authors:** Mariem Chaâbane, Mohamed Koubaa, Nejla Soudani, Awatef Elwej, Malek Grati, Kamel Jamoussi, Tahia Boudawara, Semia Ellouze Chaabouni, Najiba Zeghal

**Affiliations:** a Animal Physiology Laboratory, Department of Life Sciences, University of Sfax, Sciences Faculty of Sfax, Sfax, Tunisia;; b Enzymes and Bioconversion Unit, Department of Biological Engineering, National Engineering School of Sfax, University of Sfax, Sfax, Tunisia;; c Department of Industrial Process Engineering, Integrated Transformations of Renewable Material Unit, Research Center of Royallieu, Sorbonne University, Compiegne University of Technology, Compiegne Cedex, France;; d Biochemistry Laboratory, Department of Biochemistry, University of Sfax, CHU Hedi Chaker, Sfax, Tunisia;; e Histopathology Laboratory, Department of Anatomo-pathology, University of Sfax, CHU Habib Bourguiba, Sfax, Tunisia

**Keywords:** Triazole fungicides, nephrotoxicity, nitrariaceae, polyphenols

## Abstract

**Context:***Nitraria retusa* (Forssk.) Asch. (Nitrariaceae) is a medicinal plant which produces edible fruits whose antioxidant activity has been demonstrated.

**Objective:** The current study elucidates the potential protective effect of *N. retusa* fruit aqueous extract against nephrotoxicity induced by penconazole, a triazole fungicide, in the kidney of adult rats.

**Materials and methods:** Adult Wistar rats were exposed either to penconazole (67 mg/kg body weight), or to *N. retusa* extract (300 mg/kg body weight) or to their combination. Penconazole was administered by intra-peritoneal injection every 2 days from day 7 until day 15, the sacrifice day, while *N. retusa* extract was administered daily by gavage during 15 days. Oxidative stress parameters, kidney biomarkers and histopathological examination were determined.

**Results:***Nitraria retusa* extract administration to penconazole treated rats decreased kidney levels of malondialdehyde (−10%), hydrogen peroxide (−12%), protein carbonyls (PCOs, −11%) and advanced oxidation protein products (AOPP, −16%); antioxidant enzyme activities: catalase (−13%), superoxide dismutase (−8%) and glutathione peroxidase (GPx, −14%), and the levels of non-enzymatic antioxidants: non-protein thiols (−9%), glutathione (−7%) and metallothionein (−12%). Furthermore, this plant extract prevented kidney biomarker changes by reducing plasma levels of creatinine, urea, uric acid and LDH and increasing those of ALP and GGT. Histopathological alterations induced by penconazole (glomeruli fragmentation, Bowman’s space enlargement, tubular epithelial cells necrosis and infiltration of inflammatory leucocytes) were attenuated following *N. retusa* administration.

**Discussion and conclusion:** Our results indicated that *N. retusa* fruit extract had protective effects against penconazole-induced kidney injury, which could be attributed to its phenolic compounds.

## Introduction

During the last decades, the use of pesticides has increased dramatically, worldwide, in an effort to increase food production and control vector-borne diseases. Nevertheless, these agrochemicals have been claimed to be harmful to the environment and to the human health (Hou & Wu 2010). Indeed, pesticides are released in large amounts in the environment, leading consequently to contamination of water, soil and air. Their residues have also been detected in food products prepared for human consumption (Cabras & Angioni 2000). The World Health Organization (WHO) estimates that there are 3 million cases of pesticides poisoning in each year and up to 220,000 deaths. Moreover, epidemiological studies have stated a causal connection between human exposure to pesticides and a number of health outcomes including endocrine disrupting effects (Goetz et al. 2007), mutagenicity (Bhinder & Chaudhry 2013), neurotoxicity (Galal et al. 2014), carcinogenicity (Greim et al. 2015), hepatotoxicity (Ogutcu et al. 2008), and nephrotoxicity (Hou et al. 2014).

Among pesticides, penconazole [1-(2-(2,4-dichlorophenyl)-pentyl)-1H-1,2,4-triazole], a systemic triazole fungicide with preventive and curative properties, is widely and effectively used throughout the world for the prevention and control of powdery mildew disease in vineyards and other crops. This fungicide belongs to conazoles, a class of azole-based fungicides widely used in agriculture as well as in human and veterinary medicine applications for the treatment of local and systemic fungal infections. It acts by inhibiting a specific cytochrome P450, lanosterol-14α-demethylase (CYP51), which is required for ergosterol biosynthesis in order to maintain the proper membrane fluidity and permeability in fungi (Zarn et al. 2003). Penconazole is classified by WHO as Class U to present, unlikely, an acute hazard in normal use. In a previous study, this fungicide has been shown to be toxic to the reproductive system of male rats (El-Sharkawy & El-Nisr 2013). In addition, we have recently demonstrated that penconazole is a potent hepatotoxic and cardiotoxic (Chaâbane et al. 2015, 2016) fungicide in rats. Furthermore, toxicological studies have shown an extensive oral absorption, a wide distribution in the body and an excretion of this fungicide predominately via urine, where numerous metabolites have been identified.

Since the kidney is the major organ for xenobiotics release, renal cells are thus exposed to high doses of metabolites, causing nephrotoxicity. So, kidney is considered as a sensitive organ against pesticides-induced toxicity and damage (Kalendar et al. 2007). Indeed, various pesticides have been demonstrated to cause renal damage (Ahmed et al. 2010; Shah & Iqbal 2010; Ben Amara et al. 2013). Oxidative stress and reactive oxygen species (ROS) production have been reported as the main mechanism of the nephrotoxicity (Shah & Iqbal 2010). ROS, when produced in excess, could damage critical molecules such as lipids, proteins and nucleic acid bases, thereby resulting in cell cycle arrest and apoptosis (Perry et al. 2001; Barnham et al. 2004; Ting et al. 2010).

To counteract oxidative stress, endogenous and exogenous antioxidants play a crucial role to remove ROS. They act as free radical scavengers preventing cells and tissue damage. Exogenous antioxidants obtained from natural sources are considered to be relatively safe and without undesirable side effects (Xavier et al. 2004). Medicinal plants and herbs constitute a promising source of natural antioxidants. Many plant extracts are endowed with a variety of free radical scavenging molecules. Among them, phenolic compounds are well-known for their antioxidative activity, attributed mainly to their redox properties allowing them to act as the reducing agents, the hydrogen donors, the singlet oxygen quenchers as well as the metal chelators (Rice-Evans et al. 1995).

*Nitraria retusa* (Forssk.) Asch. (Nitrariaceae), a native perennial species, is a salt-tolerant and drought-resistant shrub, which produces fleshy red fruits eaten by humans (Le Floc’h 1983). A tasty refreshing juice may be extracted from the mature fruits. This plant is distributed in North Africa including Tunisia. Commonly known as ‘Ghardaq’, *N. retusa* is used in traditional medicine. The dry leaves serve as supplement to tea and used to make cataplasms. The ashes of this plant are applied on infected wounds in order to remove liquids (blood, lymph) and to facilitate the healing process (Shaltout et al. 2003). We have previously shown that the aqueous extract obtained from *N. retusa* fruit can serve as a source of bioactive compounds such as polyphenols and flavonoids (Chaâbane et al. 2014). We have also demonstrated that this extract exerts *in vitro* (Chaâbane et al. 2014) as well as *in vivo* (Chaâbane et al. 2015), a significant antioxidant activity. Nevertheless, the phenolic composition of *N. retusa* fruit has not been previously described.

To the best of our knowledge, no studies have examined the potential penconazole-induced nephrotoxicity. Therefore, the present study was conducted to assess some biochemical parameters, antioxidant status and histopathological alterations in the kidney of penconazole-exposed rats. We tried also to identify, for the first time, the phenolic compounds present in the aqueous extract of *N. retusa* fruit and to determine whether the treatment with this extract could alleviate penconazole-induced nephrotoxicity.

## Materials and methods

### Chemicals

Penconazole is a triazole fungicide (C_13_H_15_Cl_2_N_3_). The commercial formulation studied in the present work was Topas^®^, which contained 100 g/L of penconazole, the active ingredient, and was produced by Syngenta Company (Bâle, Suisse). Glutathione (GSH), 5,5′-dithiobis-2-nitrobenzoic acid (DTNB), thiobarbituric acid, 1,1,3,3-tetrathoxypropane (TEP), 2,4-dinitrophenylhydrazine (DNPH) and nitro blue tetrazolium (NBT) were purchased from Sigma Chemical Co. (St. Louis, MO). The other ones of analytical grade were purchased from standard commercial suppliers.

### Plant material

Fresh ripe fruits of *N. retusa* were collected from Kerkennah Island (Sfax, Tunisia). The plant was identified in botanic laboratory (Faculty of Sciences, University of Sfax, Tunisia), according to the Flora of Tunisia (Pottier-Alapetite 1979).

### Mineral composition of *N. retusa* fruit

The content of magnesium (Mg^2+^), sodium (Na^+^), calcium (Ca^2+^), zinc (Zn^2+^), iron (Fe^2+^) and copper (Cu^2+^) in *N. retusa* fruit was determined by atomic absorption spectrometry (Sherwood 410) after digestion with concentrated nitric acid (AOAC 2000). Briefly, after being separated from the seeds, the fruit pulps of *N. retusa* were homogenized in a domestic blender (Ufesa Supplier, Spain). An amount of 1 g of the homogenate was dried at 105 °C for 24 h and subsequently incinerated in a muffle furnace at 550 °C for 4 h. The residue of incineration was cooled, dissolved in 1 mL of concentrated nitric acid and allowed to stand for 30 min. After that, the sample was diluted with 10 mL of deionized water and filtered with Whatman No.1 filter paper. Then, the solution was transferred to a volumetric flask and diluted to a final volume of 25 mL with deionized water. The resulting extract was used for mineral content determination. Results were expressed as mg/100 g *N. retusa* fresh weight (NRFW).

### Preparation of *N. retusa* fruit aqueous extract

An aqueous extract of *N. retusa* fruit was prepared following the procedure of Benvenuti et al. (2004), with slight modifications. Briefly, 200 g of the previously prepared fruit homogenate were extracted with 200 mL distilled water under continuous stirring for 60 min. The extract was filtered under vacuum through Whatman No.1 filter paper on a Buchner funnel, and the residue was extracted again by the same way. The obtained filtrates were combined, lyophilized and kept in the dark at 4 °C until use.

### High-pressure liquid chromatography–high resolution mass spectrometry (HPLC-HRMS) analysis

The phenolic compounds present in the aqueous extract of *N. retusa* fruit were analyzed using ultra high-pressure liquid chromatography (UPLC 1290 Infinity) coupled with high resolution–mass spectrometry (HR-MS Q-TOF UHD 6538) from Agilent Technologies. In brief, chromatographic separation was carried out using a Thermo Hypersyl Gold column (4.6 × 150 × 5 μm) at a flow rate of 0.7 mL/min. The separation gradient was generated using 0.1% formic acid in water (solvent A), and acetonitrile (solvent B). The gradient was defined as follows: from 0 to 20 min, 6% B; from 20 to 35 min, 18% B; from 35 to 45 min, 28% B; from 45 to 48 min, 60% B; from 48 to 55 min, 90% B. Cleaning of the column was achieved with 90% B for 5 min. The mass spectra were acquired using a dual ESI ionization in positive-ion mode. The nebulization gas and the ion spray voltage were adjusted to 30 psi and 3.5 kV, respectively. The temperature of the ion source was fixed to 300 °C. All data were acquired and processed using Mass Hunter B.05.01 software. The identification was performed according to a home-generated database containing 500 compounds and a mixture of standards containing 30 phenolic compounds, as described by Koubaa et al. (2015).

### Animals and treatment

A total of 24 male Wistar rats (251 ± 3 g) were purchased from the Central Pharmacy (SIPHAT, Tunisia). The rats were housed under standard laboratory conditions of temperature (22 ± 2 °C), relative humidity (40%) and 12 h light/dark cycles. They were fed with standard pellet diet (SNA, Sfax, Tunisia) and given drinking water *ad libitum*. Approval for rat experiments was obtained from the ethical Committee at Sciences Faculty of Sfax with ethics approval number 1204 and all the experimental procedures were in accordance with the International Guidelines for Animal Care (Council of European Communities 1986). The animals were randomly divided into four groups of six each as follows:

- Group 1 (Controls): rats received distilled water.
- Group 2 (PEN): no treatment was performed during the first 6 days, then rats received intraperitoneally 67 mg/kg body weight (1/30 LD50) of penconazole every 2 days from day 7 until day 15, the sacrifice day.
- Group 3 (NRE + PEN): received daily by gavage during the first six days 300 mg/kg body weight of lyophilized *N. retusa* extract (corresponding, for each rat weighing a mean value of 251 g, to 75 mg of lyophilized *N. retusa* extract dissolved in 1 mL of distilled water). From day 7 until day 15, rats received aqueous extract of *N. retusa* fruit (300 mg/kg body weight) daily by gavage and penconazole (67 mg/kg body weight) every 2 days by intraperitoneal way.
- Group 4 (NRE): serving as positive controls, received daily by gavage *N. retusa* aqueous extract (300 mg/kg body weight) for 15 days.

The dose of penconazole used in the present work was selected on the basis of the previous study carried out by El-Sharkawy and El-Nisr (2013). These authors have used two doses of penconazole, 50 mg/kg body weight and 100 mg/kg body weight (representing, respectively, 1/40 and 1/20 of LD_50_), which induce structural and functional testicular impairment in adult male rats. For our experiment, we have chosen an intermediate dose (67 mg/kg body weight), which represented 1/30 LD_50_ and caused toxicity without lethality. Indeed, a dose higher than 67 mg/kg body weight provoked haemorrhage and diarrhoea. Concerning *N. retusa* extract, a preliminary dose-response study showed that a dose higher than 300 mg/kg body weight was found to cause diarrhoea in exposed rats, which could be due to the presence of fibres in *N. retusa* fruits as reported by Hegazy et al. (2013).

Twenty four hours before the sacrifice day, the rats were placed individually in metabolic cages for urine collection. After recording 24 h urine volumes, samples were centrifuged at 5000 *g* for 5 min to eliminate the probable presence of excrements. The supernatants were collected and then stored at −20 °C for further analysis. At the end of the experiment and after 15 days of treatment, the rats were sacrificed by cervical dislocation to avoid stress condition. Blood samples were collected into heparin-coated tubes and centrifuged at 2200 *g* for 10 min. Plasma samples were drawn and stored at −20 °C until analysis. Kidney tissues were dissected out, cleaned from adipose tissue and weighed. Some samples were rinsed, homogenized in Tris-HCl buffer (pH 7.4) and centrifuged. The resulting supernatants were collected and kept at −80 °C until biochemical analysis. Other samples were fixed in 10% buffered formalin solution and embedded in paraffin for histological examination. For the biochemical and the histological experiments, samples (kidney tissue, plasma, urine) were taken from six rats in each group.

### Biochemical assays

#### Protein quantification in kidneys

Kidney protein content was determined by Lowry et al. (1951) method using bovine serum albumin as standard.

#### Estimation of urea, uric acid, creatinine and creatinine clearance

The levels of urea, uric acid and creatinine in plasma and urine were assayed spectrophotometrically using commercially available diagnostic kits (Biomaghreb, Tunisia, Ref. 20143, 20095, 20151, respectively). Creatinine clearance was calculated using *UV*/*P* equation (Charrel 1991), where *U* is the urinary creatinine level, *V* the urinary volume collected within 24 h and *P* the plasma creatinine level.

#### Kidney and plasma lactate dehydrogenase activities

Lactate dehydrogenase (LDH) activities in the kidney and plasma were measured using a commercially available diagnostic kit (Biomaghreb, Tunisia, Ref. 200125).

#### Plasma alkaline phosphatase and gamma glutamyltranspeptidase activities

Plasma alkaline phosphatase (ALP) and gamma glutamyltranspeptidase (GGT) activities were determined using diagnostic kits (Biomaghreb, Tunisia, Ref. 20015 and 20022, respectively).

#### Evaluation of kidney lipid peroxidation

The concentrations of malondialdehyde (MDA), an index of lipid peroxidation, in kidney tissue were determined according to the method by Draper and Hadley (1990). The MDA values were calculated using TEP as standard and expressed as nmol of MDA/g tissue.

#### Estimation of hydrogen peroxide generation

Hydrogen peroxide (H_2_O_2_) generation in kidney tissue was assessed by Ou and Wolff method (Ou & Wolff 1996). The amount of H_2_O_2_ in the sample was determined using the extinction coefficient of 2.67 × 10^5^ cm^−1^ M^−1^ and results were expressed as nmol/mg protein.

#### Determination of protein carbonyl levels in kidney

Protein carbonyl (PCO) content in kidney tissue was measured using the DNPH method by Reznick and Packer (1994). Results were expressed as nmol/mg protein.

#### Determination of advanced oxidation protein product levels in kidney

The kidney levels of advanced oxidation protein products (AOPP) were determined according to the method of Kayali et al. (2006), using the extinction coefficient of 261 cm^−1 ^mM^−1^. Results were expressed as nmol/mg protein.

#### Determination of antioxidant enzyme activities

The catalase (CAT) activity was assayed as described by Aebi (1984) using H_2_O_2_ as substrate. The decrease in absorbance due to H_2_O_2_ degradation was recorded at 240 nm and CAT activity was calculated in terms of μmol H_2_O_2_ consumed/min/mg of protein.

The superoxide dismutase (SOD) activity was determined by monitoring the photochemical reduction of NBT according to the method of Beauchamp and Fridovich (1971). One unit of SOD activity corresponded to the amount of enzyme required to cause 50% inhibition of NBT reduction at 560 nm. SOD activity was expressed as units/mg protein.

The glutathione peroxidase (GPx) activity was estimated according to the method of Flohe and Gunzler (1984). The enzyme activity was expressed as nmol of GSH oxidized/min/mg protein.

#### Determination of non-protein thiol levels

Kidney non-protein thiol (NPSH) levels were determined by the method by Ellman (1959) and the results were expressed as μmol/g tissue.

#### Determination of GSH levels

The GSH content in kidney tissue was assayed according to the method by Ellman (1959) modified by Jollow et al. (1974). Results were expressed as μg/g tissue.

#### Determination of metallothionein content

The concentration of metallothionein (MT) in kidney tissue was estimated according to the method by Viarengo et al. (1997) modified by Petrovic et al. (2001). Results were expressed as μmol GSH/g tissue.

### Histological studies

For histological examination, some kidneys were removed from control and treated rats. They were fixed in 10% buffered formalin solution and then embedded in paraffin. Sections of 5 μm thickness were placed on slides and stained with haematoxylin and eosin for histological evaluation under light microscopy. Six slides were prepared from each kidney tissue. All sections were evaluated for the degree of tubular and glomerular injury. Each kidney slide was examined and assigned for severity of changes using scores on a scale of none (−), mild (+), moderate (++) and severe (+++) damage.

### Statistical analysis

The data were analyzed using the statistical package program Stat view 5 Software for Windows (SAS Institute, Berkley, CA). Statistical analysis was performed using one-way analysis of variance (ANOVA) followed by Fisher protected least significant difference (PLSD) test as a *post hoc* test for comparison between groups. Student unpaired *t* test, used for comparison between two groups, was also required. All values were expressed as means ± SD. Differences were considered significant if *p* < 0.05.

## Results

### *In vitro* study

#### Mineral analysis of *N. retusa* fruit

The mineral content of *N. retusa* fruit was determined in the present study (Table 1). The most abundant macro-element was Na^+ ^(410.82 mg/100 g NRFW) while Ca^2+ ^and Mg^2+ ^were found in smaller amounts than Na^+ ^(101.82 and 54.69 mg/100 g NRFW, respectively). Regarding the microelements, the highest value was represented by Fe^2+ ^(6.78 mg/100 g NRFW), followed by Zn^2+ ^(0.48 mg/100 g NRFW) and Cu^2+ ^(0.322 mg/100 g NRFW).

**Table 1.** Mineral content of *N. retusa* fruit.

Minerals	Concentration (mg/100 g*N. retusa* fresh weight)
Mg^2+^	54.69
Na^+^	410.82
Ca^2+^	101.82
Zn^2+^	0.48
Fe^2+^	6.78
Cu^2+^	0.322

Mg^2+^: magnesium, Na^+^: sodium, Ca^2+^: calcium, Zn^2+^: zinc, Fe^2+^: iron, Cu^2+^: copper.

#### Polyphenols identification

**Figure 1.**
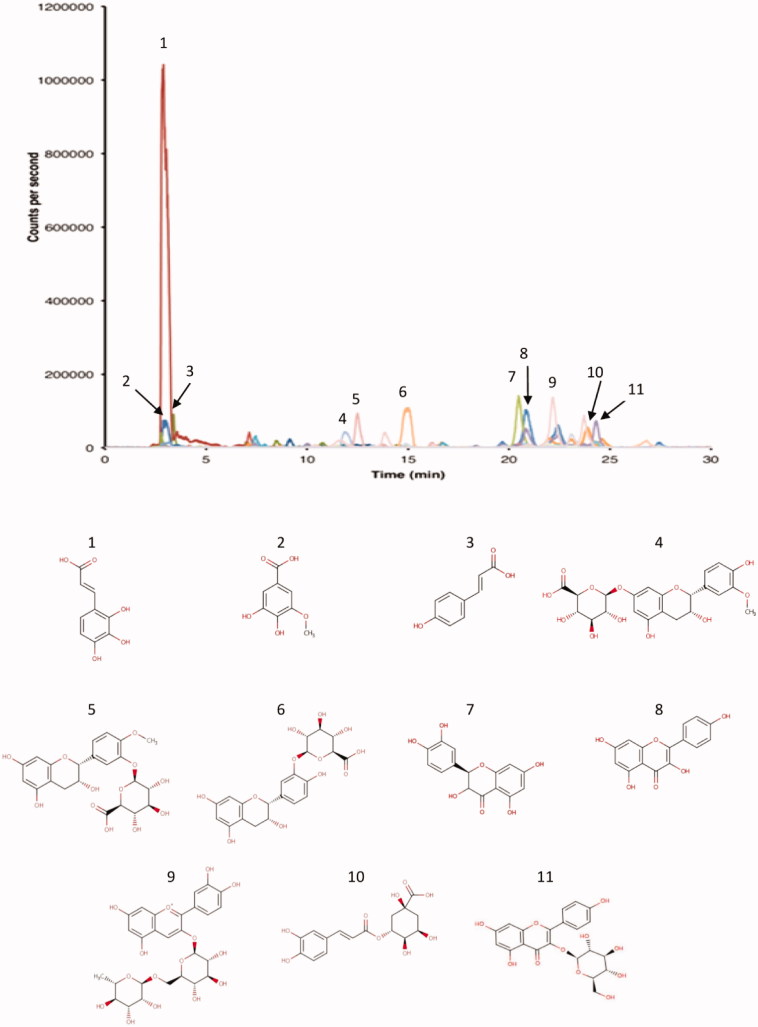
HPLC–HRMS chromatogram representing the polyphenols extracted from the aqueous extract of *N. retusa* fruit and chemical structure of the major components identified [hydroxycaffeic acid (**1**), 3-*O*-methylgallic acid (**2**), *p*-coumaric acid (**3**), 3′-*O*-methyl-(-)-epicatechin 7-*O*-glucuronide (**4**), 4′-*O*-methyl-(-)-epicatechin 3′-*O*-glucuronide (**5**), epicatechin 3′-*O*-glucuronide (**6**), taxifoline (**7**), kaempferol (**8**), cyanidin 3-*O*-rutinoside (**9**), chlorogenic acid (**10**) and kaempferol 3-glucoside (**11**)]. The identification was performed according to a home-generated database containing 500 compounds and a mixture of standards containing 30 phenolic compounds.

The phenolic compounds profile of *N. retusa* fruit aqueous extract is shown in Figure 1. Based on analysis by HPLC-HRMS, the major identified components were hydroxycaffeic acid **(1)**, 3-*O*-methylgallic acid **(2)**, *p*-coumaric acid **(3)**, 3′-*O*-methyl-(-)-epicatechin 7-*O*-glucuronide **(4)**, 4′-*O*-methyl-(-)-epicatechin 3′-*O*-glucuronide **(5)**, epicatechin 3′-*O*-glucuronide **(6)**, taxifoline **(7)**, kaempferol **(8)**, cyanidin 3-*O*-rutinoside **(9)**, chlorogenic acid **(10)** and kaempferol 3-glucoside **(11)**.

### *In vivo* study

#### Body, absolute and relative kidney weights. Food and water intakes

During the experimental period, there was no observed mortality in any experimental group. Moreover body, absolute and relative kidney weights as well as the daily food intake of treated animals were similar to those of controls. While a significant increase (+30%) in the daily water consumption was shown in penconazole-treated rats, as compared to the controls (Table 2).

**Table 2.** Body, absolute and relative kidney weights. Food and water intake of control and treated rats with penconazole (PEN), *N. retusa* aqueous extract (NRE), or their combination (NRE + PEN).

Parameters and treatments	Controls	PEN	NRE + PEN	NRE
Initial body weight (g)	248.67 ± 8.48	249.67 ± 5.61	251.67 ± 4.27	254.17 ± 6.43
Final body weight (g)	261.00 ± 6.81	264.33 ± 5.20	262.00 ± 4.86	267.67 ± 9.18
Absolute kidney weight (g)	1.61 ± 0.15	1.68 ± 0.10	1.61 ± 0.06	1.76 ± 0.17
Relative kidney weight (g/100g bw)	0.62 ± 0.05	0.63 ± 0.03	0.61 ± 0.02	0.66 ± 0.05
Food intake (g/day/rat)	16.68 ± 1.59	16.08 ± 1.77	15.89 ± 1.55	16.39 ± 1.42
Water intake (mL/day/rat)	25.47 ± 2.45	33.16 ± 2.64***	26.07 ± 2.22^###^	26.03 ± 1.64

Values are means ± SD for six rats in each group.

bw: body weight.

PEN group vs. control group:

*** *p* < 0.001.

NRE + PEN group vs PEN group:

### *p* < 0.001.

#### Biomarkers of renal toxicity

Results showed that creatinine, urea and uric acid levels of penconazole-treated rats were higher in plasma (+20, +61 and +65%, respectively) and lower in urine (−71, −66 and −47%) than those of the controls (Table 3). These observations were associated with a significant elevation of 24 h urinary volume and a reduction in creatinine clearance (Table 3). Treatment of penconazole-treated rats with *N. retusa* extract improved the levels of renal biomarkers, compared to those of PEN group.

**Table 3.** Urinary volume, creatinine clearance, plasma and urinary levels of creatinine, urea and uric acid, and BUN of control and treated rats with penconazole (PEN), *N. retusa* aqueous extract (NRE) or their combination (NRE + PEN).

Parameters and treatments	Controls	PEN	NRE + PEN	NRE
Urinary volume (mL/24 h)	5.33 ± 0.41	14.00 ± 1.87***	11.67 ± 0.41***##	7.67 ± 1.08***
Creatinine clearance (μL/min)	536.75 ± 81.31	339.58 ± 39.50***	533.28 ± 88.10###	640.26 ± 83.20*
Creatinine (μmol/L)				
Plasma	52.40 ± 3.12	62.62 ± 6.12**	54.67 ± 4.86#	54.34 ± 0.96
Urine	7551.25 ± 573.05	2216.67 ± 440.08***	3580.63 ± 526.25***###	6550.00 ± 39.53**
Urea (mmol/L)				
Plasma	5.82 ± 0.39	9.35 ± 0.48***	7.38 ± 0.25***###	6.49 ± 0.23**
Urine	472.61 ± 66.84	161.22 ± 9.60***	234.16 ± 31.28***###	359.57 ± 19.09**
Uric acid (μmol/L)				
Plasma	73.13 ± 6.04	120.71 ± 9.60***	64.53 ± 5.30***#	76.93 ± 3.70
Urine	247.50 ± 39.47	131.67 ± 14.72***	147.5 ± 7.58***#	215.00 ± 30.17
BUN^a^ (mmol/L)	2.72 ± 0.18	4.37 ± 0.22***	3.45 ± 0.12***###	3.06 ± 0.11**

Values are means ± SD for six rats in each group.

BUN: blood urea nitrogen.

a BUN was calculated using the formula conversion:

BUN (mmol/L) = [urea(mmol/L)]/2.14.

PEN, NRE + PEN and NRE groups vs. control group:

* *p* < 0.05.

** *p* < 0.01.

*** *p* < 0.001.

NRE + PEN group vs PEN group:

# *p* < 0.05.

## *p* < 0.01.

### *p* < 0.001.

#### Plasma ALP, GGT and LDH activities and kidney LDH activity

In penconazole-treated rats, plasma ALP and GGT activities decreased by 22 and 35%, respectively, when compared to controls (Table 4). In addition, LDH activity increased by 57% in the plasma while it decreased by 22% in the kidney of penconazole-treated rats, when compared to the controls (Table 4). Administration of *N. retusa* extract increased the activities of ALP and GGT by 8 and 28%, respectively, as compared to penconazole-treated rats. Moreover, in NRE + PEN group, LDH activity decreased by 30% in the plasma and increased by 13% in the kidney, as compared to PEN group (Table 4).

**Table 4.** Plasma ALP, GGT and LDH activities and kidney LDH activity in control and treated rats with penconazole (PEN), *N. retusa* aqueous extract (NRE) or their combination (NRE + PEN).

Parameters and treatments	Controls	PEN	NRE + PEN	NRE
Plasma				
ALP (IU/L)	122.5 ± 13.65	95.42 ± 5.94**	103.08 ± 5.60**	112.67 ± 5.49
GGT (IU/L)	11.50 ± 1.75	7.44 ± 0.50***	9.52 ± 0.84*###	12.30 ± 2.43
LDH (IU/L)	1162.33 ± 97.84	1824.17 ± 94.96***	1274.67 ± 89.08*###	1312.00 ± 21.43**
Kidney				
LDH (IU/L)	3.37 ± 0.29	2.63 ± 0.23***	2.97 ± 0.29*#	3.27 ± 0.22

Values are means ± SD for six rats in each group.

ALP: alkaline phosphatase, GGT: gamma glutamyltranspeptidase, LDH: Lactate dehydrogenase.

PEN, NRE + PEN and NRE groups vs. control group:

* *p* < 0.05.

** *p* < 0.01.

*** *p* < 0.001.

NRE + PEN group vs PEN group:

# *p* < 0.05.

### *p* < 0.001.

#### Lipid peroxidation in the kidney

Our results revealed an increase of lipid peroxidation in the kidney of penconazole-treated rats as evidenced by the enhanced MDA level (+67%) when compared to controls. In NRE + PEN group, the MDA level was significantly reduced (−10%) compared to the PEN group (Table 5).

**Table 5.** Kidney MDA, H_2_O_2_, PCO, AOPP, NPSH, GSH and MT levels in control and treated rats with penconazole (PEN), *N. retusa* aqueous extract (NRE) or their combination (NRE + PEN).

Parameters and treatments	Controls	PEN	NRE + PEN	NRE
MDA (nmol/g tissue)	142.75 ± 9.63	238.01 ± 18.62***	213.81 ± 15.14***#	147.22 ± 16.49
H_2_O_2_ (nmol/mg protein)	0.042 ± 0.004	0.064 ± 0.005***	0.056 ± 0.005***#	0.045 ± 0.004
PCO (nmol/mg protein)	0.59 ± 0.05	0.84 ± 0.05***	0.75 ± 0.06***#	0.56 ± 0.05
AOPP (nmol/mg protein)	0.20 ± 0.02	0.37 ± 0.02***	0.31 ± 0.01***###	0.21 ± 0.02
NPSH (μmol/g tissue)	2.40 ± 0.12	2.76 ± 0.05***	2.50 ± 0.04*###	2.47 ± 0.07
GSH (μg/g tissue)	29.74 ± 1.45	34.05 ± 2.30**	31.55 ± 1.08*#	30.86 ± 0.71
MT (μmol GSH/g tissue)	0.49 ± 0.01	0.57 ± 0.06**	0.50 ± 0.03#	0.47 ± 0.04

Values are means ± SD for six rats in each group.

MDA: malondialdehyde, H_2_O_2_: hydrogen peroxide, PCO: protein carbonyls, AOPP: advanced oxidation protein products, NPSH: non-protein thiols, GSH: reduced glutathione MT: metallothionein.

PEN and NRE + PEN groups vs. control group:

* *p* < 0.05.

** *p* < 0.01.

*** *p* < 0.001.

NRE + PEN group vs PEN group:

# *p* < 0.05.

### *p* < 0.001.

#### Hydrogen peroxide generation in the kidney

The level of H_2_O_2_ generated in kidney was significantly increased (+52%) in the PEN group when compared to controls. Administration of *N. retusa* extract to penconazole-treated rats decreased significantly (−12%) H_2_O_2_ level as compared to the PEN group.

#### Protein oxidative markers in the kidney

In the PEN group, a significant increase of PCO and AOPP levels in the kidney tissue of adult rats (+42 and +85%, respectively) was observed when compared to the controls. Treatment with *N. retusa* extract resulted in a marked decrease of kidney PCO and AOPP levels (−11 and −16%, respectively) when compared to the Pen group (Table 5).

#### Non-enzymatic antioxidant status in the kidney

The levels of non-enzymatic antioxidants such as NPSH, GSH and MT were determined in the kidney of experimental animals. Results showed that penconazole treatment increased significantly the levels of NPSH (+15%), GSH (+16%) and MT (+16%), as compared to controls (Table 5). These modifications were alleviated following administration of *N. retusa* extract to penconazole treated rats as indicated by the significant decrease of NPSH (−9%), GSH (−7%) and MT (−12%) levels compared to those of PEN group.

#### Enzymatic antioxidant status in the kidney

**Figure 2.**
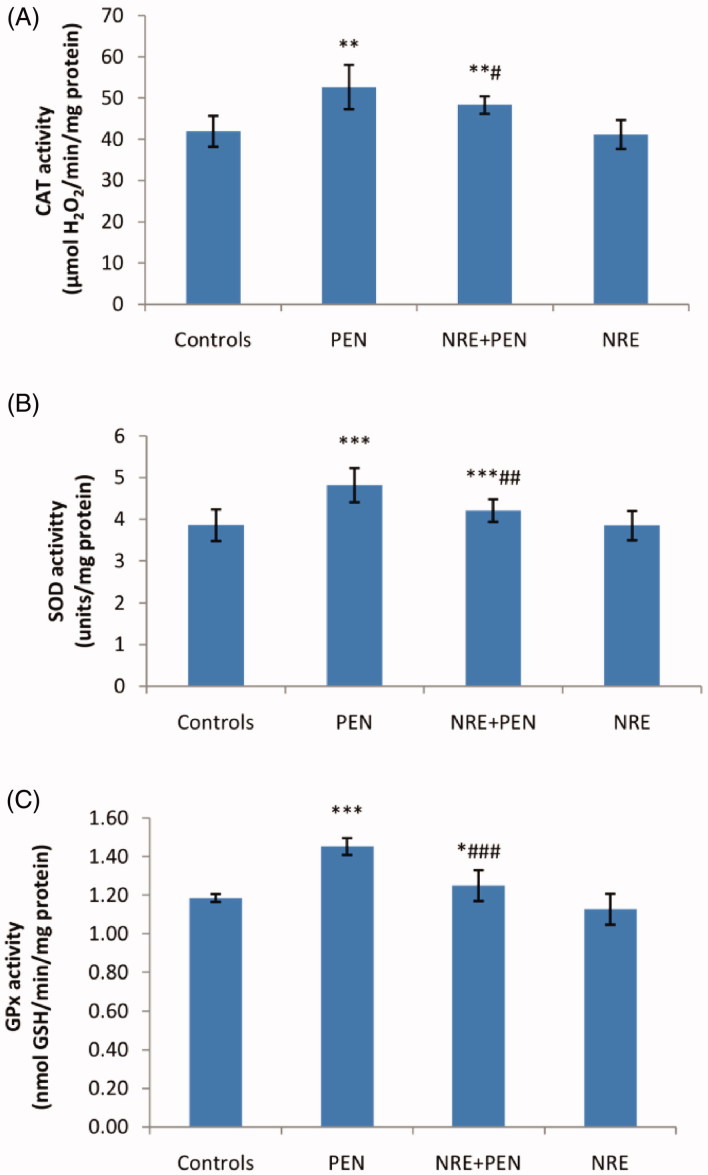
Antioxidant enzyme activities (A) CAT, (B) SOD and (C) GPx in kidney of control and treated rats with penconazole (PEN), *N. retusa* aqueous extract along with penconazole (NRE + PEN) and *N. retusa* aqueous extract (NRE). Values are means ± SD for six rats in each group. PEN and NRE + PEN groups vs control group: *p < 0.05; **p < 0.01; ***p < 0.001. NRE + PEN group vs PEN group: #*p* < 0.05; ##*p* < 0.01; ###*p* < 0.001

Penconazole treatment resulted in a significant increase of CAT (+26%), SOD (+25%) and GPx activities (+23%) when compared to controls. Treatment with *N. retusa* aqueous extract reduced significantly the activities of these enzymes by 8, 13 and 14%, respectively, compared to those of penconazole-treated group. Treatment of adult rats with *N. retusa* extract alone produced no changes in the activities of these enzymes, when compared to those of negative controls (Figure 2).

#### Kidney histoarchitecture

**Figure 3.**
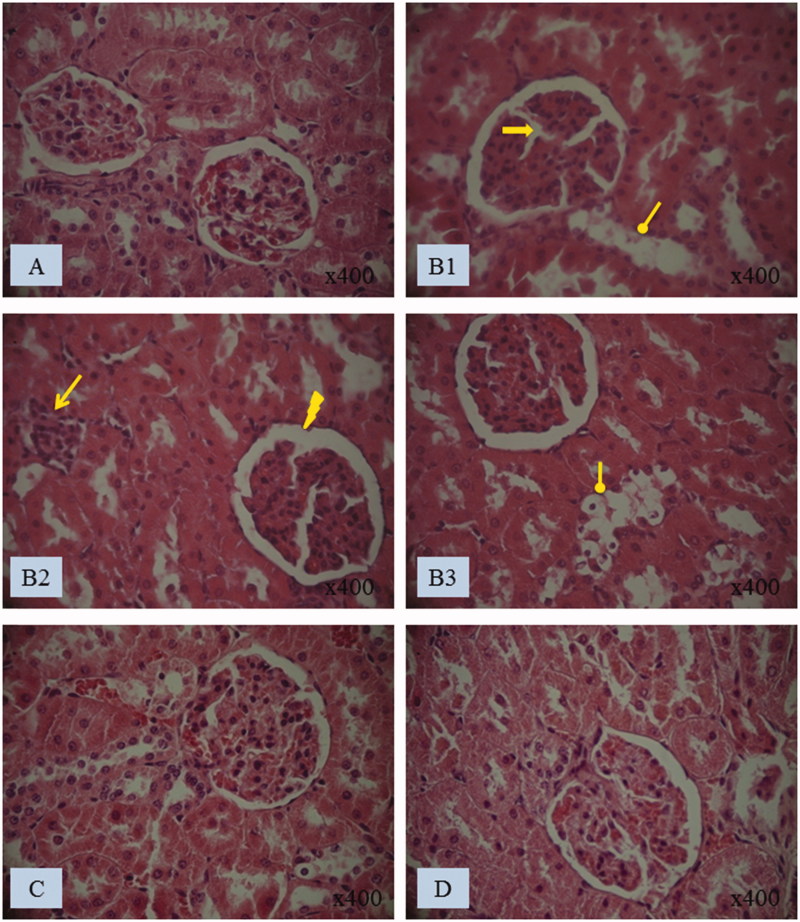
Histological kidney sections of (A) control and (B1, B2 and B3) treated rats with penconazole, (C) *N. retusa* aqueous extract along with penconazole and (D) *N. retusa* aqueous extract. Optic microscopy: H&E (400×). Arrows indicate: Glomeruli fragmentation, necrosis of the epithelial cells lining the tubules, Bowman’s space enlargement, inflammatory leucocytes infiltration

Histological studies showed that control rats presented a normal kidney histoarchitecture with normal tubules and intact glomeruli (Figure 3(A)). While kidney histology of the PEN group revealed several abnormalities, detected both in the tubules and in glomeruli, when compared to the controls. In fact, penconazole treatment caused necrosis of the epithelial cells lining the tubules (Figure 3(B1,B3)), in addition to the infiltration of inflammatory leucocytes (Figure 3(B2)). Glomeruli fragmentation and Bowman’s space enlargement were also observed in the kidney of penconazole-treated rats (Figure 3(B1,B2)). The co-administration of *N. retusa* extract to penconazole-treated rats reduced markedly the glomeruli fragmentation and the Bowman’s space enlargement when compared to the PEN group (Figure 3(C)). Furthermore, this extract attenuated the infiltration of inflammatory leucocytes and the damages to the epithelial cells lining the tubules induced by penconazole (Figure 3(C)). Microscopic examination of kidney sections of NRE group showed normal histological structure of renal tubules and glomeruli (Figure 3(D)). The histological changes in treated rats were graded and summarized in Table 6.

**Table 6.** Grading of the histopathological changes in the kidney sections.

Groups	Enlarged Bowman’sspace	Glomerulifragmentation	Necrosis of the epithelial cellslining the tubules	Infiltration ofleucocytes
Controls	−	−	−	−
PEN	++	+++	+++	+++
NRE + PEN	−	−	+	−
NRE	−	−	−	−

Scoring was done as follows: none (−), mild (+), moderate (++) and severe (+++).

## Discussion

In recent years, there is a growing concern worldwide over the indiscriminate use of pesticides, resulting in environmental pollution and toxicity risk to non-target organisms. Indeed, pesticides exposure has been associated with free radical-mediated damage in biological systems (Banerjee et al. 2001). This has prompted the intensive efforts to search for new antioxidant compounds, especially from natural sources, which are considered to be relatively safe and without side effects. Medicinal plants have been largely used in traditional medicine as remedies for many kinds of human diseases. The present study was undertaken to evaluate the potential toxic effects of penconazole on the kidney of adult rats and to investigate the possible protective effect of *N. retusa* fruit aqueous extract against toxicity induced by this fungicide.

In the present work, penconazole treatment had no effect on body weight as well as on food intake of adult rats, suggesting that this fungicide did not probably affect their appetite. Absolute and relative kidney weights were also unaffected following penconazole exposure. While, a significant rise in water consumption was recorded for penconazole-treated rats, which could be explained as a mechanism to counteract the toxicity induced by this fungicide.

Although there were no morphological changes including body or kidney weights in penconazole-treated rats, exposure to this fungicide caused alteration of renal function. Indeed, creatinine, creatinine clearance, urea and blood urea nitrogen (BUN) are used as indicators of renal function. Our results showed an increase of plasma levels in creatinine, urea, BUN and a reduced creatinine clearance in penconazole-treated rats, indicating renal dysfunction with a reduction in glomerular filtration rate. Increased plasma creatinine and BUN levels have been recorded by Takaori (1993) in rats fed with thiophanate-methyl, a benzimidazole fungicide, and by Badgujar et al. (2014) in rats exposed to fipronil, a phenyl pyrazole insecticide. This impairment in glomerular filtration rate was accompanied by an increase in 24-h urine volume, which might happen as a consequence of a reduced water reabsorption capacity by tubular epithelial cells.

Uric acid was another parameter used in the present work to assess kidney function. It is known for its antioxidant capacity based on its ability to scavenge free radicals (Alvarez-Lario & Macarron-Vicente 2010). An increase of plasma uric acid level after exposure to penconazole might reflect body response to an excess of ROS production, since uric acid is a potent free radicals scavenger. This finding is in agreement with the previous study of El-Demerdash et al. (2013) where plasma uric acid was found to be increased in the kidney of rats exposed to methomyl, a carbamate pesticide.

Administration of *N. retusa* aqueous extract to penconazole-treated rats attenuated the kidney impairment as suggested by a significant amelioration of the renal biomarkers indicated earlier. Nevertheless, we have noted a significant increase of urinary volume in rats receiving *N. retusa* aqueous extract alone, as compared to control rats. Our data indicated that this wild medicinal plant exerted probably a diuretic activity which could be due to its high salt content, as reported by us. In fact, *N. retusa* fruit contained high amounts of Na^+^,^ ^which could be due to its halophyte nature. Besides, according to Hegazy et al. (2013), this species fruit is rich in potassium (263.33 ± 6.11 mg/100 g NRFW), which is reported to have a diuretic effect (Nelson & Cox 2004). In addition, the presence of flavonoids in *N. retusa* fruit (Chaâbane et al. 2014) could explain the increase of diuresis as reported by Yuliana et al. (2009) who have indicated that flavonoids cause polyuria. Similar results have been found by Durairaj et al. (2007) concerning the effects of *Oxystelma esculentum* R. Br. (*Asclepiadaceae*) methanolic extract on diuresis in rats. Treatment of rats with *N. retusa* fruit aqueous extract induced also a significant rise in plasma urea and BUN values, which could be ascribed to the dehydratation caused by the diuretic effect of this extract, as indicated by Nayeem and Quadri (2015) in albino mice administered with *Boswellia serrata* Roxb. (*Burseraceae*) leaf extract.

As described previously, kidney function impairment could be attributed to oxidative damage probably caused by penconazole. It is well established that the kidney is highly vulnerable to the damage induced by ROS due to its high content of polyunsaturated fatty acids (Rodrigo & Rivera 2002). In the present study, penconazole induced lipid peroxidation in the kidney of adult rats as revealed by a marked elevation in MDA. Previous findings of Sakr (2007) have shown that treatment with mancozeb, another fungicide, induces a significant increase in lipid peroxidation in albino rats. The increased MDA level observed in tissues of animals exposed to penconazole could be probably ascribed to the excessive production of ROS caused by this fungicide leading to renal injury. In fact, the generation of the H_2_O_2_ was enhanced following treatment of rats with penconazole. Consistent with these observations, Poovala et al. (1998) have found an increase of H_2_O_2_ levels following exposure of renal tubular cells to acephate, an organophosphate insecticide. H_2_O_2_ generation has been involved in the pathogenesis of several forms of acute tubular injury, where the lipid peroxidation process plays an essential role (Salahudeen 1995). Mitochondria are considered to be the major cellular source for H_2_O_2_ production. Hence, it is suggested that penconazole, with lipophilic nature, could easily cross the cytoplasmic membrane to reach mitochondria, causing thereby the dysfunction of the mitochondrial respiratory chain and an enhanced generation of ROS including H_2_O_2_ which could be converted to a reactive hydroxyl radical. The latter can trigger a lipid peroxidation chain reaction by extracting an allylic hydrogen from polyunsaturated fatty acids.

As a consequence of lipid peroxidation, biological membranes are affected leading to the loss of their fluidity, a modification of their potential or an increase of their permeability leading to a cellular damage. In this regard, LDH, an intracellular enzyme, is recognized to be a potent marker for assessing chemical’s toxicity. This enzyme is released into the blood stream once the cell membrane integrity is disrupted in the conditions of oxidative stress (Zou et al. 2001). Our results demonstrated that, in penconazole-treated rats, LDH activity decreased significantly in kidney tissues while it increased in plasma when compared to controls. This observation might be attributed to a generalized increase in membrane permeability as a result of lipid peroxidation under ROS generation by penconazole.

In addition to LDH, ALP and GGT, which are membrane bound enzymes, can be also used as the reliable biomarkers of cell and tissue damage induced by toxic substances. In the current study, penconazole exposure resulted in a significant decline of plasma ALP activity which might be attributed to a decreased osteoblastic activity in bone, since ALP is formed in the osteoblasts. Hence, the bone tissue could be affected by penconazole exposure. Our results showed also a significant reduction in plasma GGT activity in penconazole-treated rats which might be due to the occurrence of oxidative stress through an excessive generation of ROS, as reported by Abdelhalim and Moussa (2013) in rats exposed to gamma radiation. Likewise, Abd-Alrahman et al. (2014) have demonstrated that exposure of rats to difenoconazole, another triazole fungicide, decreases plasma GGT activity.

Moreover, ROS can also directly attack cellular proteins leading to a severe failure of their biological functions and even cell death. Among the various oxidative modifications of amino acids in proteins, PCO formation may be an early marker for protein oxidation. Indeed, the process of protein carbonylation is nonreversible, causing conformational changes, decreases enzyme catalytic activities, resulting in the breakdown of proteins by proteases (Zhang et al. 2008). In addition, the degree of protein damage in experimental rats can be estimated by a novel marker, AOPP. Under our experimental conditions, the increased PCO and AOPP levels were detected in the kidney of penconazole-treated rats suggesting that oxidative protein damage might be one of the mechanisms underlying penconazole-induced nephrotoxicity. Previous studies have shown that pesticides exposure may promote protein oxidation in the kidney of adult rats (Ben Amara et al. 2013).

Treatment with *N. retusa* aqueous extract prevented oxidative damage induced by penconazole in the renal tissue, objectified by a significant decrease in the levels of MDA, H_2_O_2_, PCO and AOPP, as well as an improvement of ALP and GGT activities. This might be attributed to the free radical scavenging property of the plant extract.

Defence of renal cells against oxidative stress is maintained by several mechanisms including enzymatic and non-enzymatic antioxidants. Antioxidant enzymes such as CAT, SOD and GPx present an impressive array of defence mechanisms, since they are effective in maintaining ROS under adequate concentrations. SOD accomplishes its antioxidant function by catalyzing the dismutation of the superoxide radical ($O_{2}^{•-}$) to H_2_O_2_, protecting thereby the kidney from $O_{2}^{•-}$ damage. The production of the potentially harmful H_2_O_2_ is counteracted by the action of CAT and GPx, catalyzing the decomposition of this ROS to water and oxygen. In the current study, penconazole treatment provoked changes in renal enzymatic antioxidant systems as confirmed by the increased activities of CAT, SOD and GPx. The increment in the activities of these enzymes might be understood as an adaptive response of the body to oxidative stress as reported by Sengupta et al. (1990) in rats exposed to chromium.

As antioxidant enzymes work in synergy with non-enzymatic antioxidants, it was necessary to evaluate renal GSH and NPSH levels. GSH is thought to be an important endogenous defence molecule against peroxidative destruction of cellular membranes. This tripeptide can react directly with ROS and electrophilic metabolites and protects essential thiol groups from oxidation. In our study, significant rises in kidney GSH and NPSH levels were noted following penconazole treatment, which might occur as a mechanism of protection against oxidative damage.

Cellular defence against oxidative stress is also induced by metallothionein (MT), a small metal-binding and cysteine-rich protein. Its high thiol content makes it a powerful OH^•^ scavenger more efficient than GSH (Deneke 2000). This protein has been widely used to identify specific responses to heavy metals pollution. MT synthesis can be also induced by compounds other than heavy metals leading to ROS production. In our study, penconazole stimulated the kidney to produce MT as illustrated by its significant increase when compared to controls. This could be considered as a defence mechanism, in addition to the antioxidants, against oxidative stress and the adverse effects induced by this fungicide.

Interestingly, treatment with *N. retusa* aqueous extract improved the enzymatic and non-enzymatic antioxidant status of rats exposed to penconazole, indicating that this plant extract participated in the reduction of penconazole toxicity by decreasing oxidative stress due to the ability of its antioxidant compounds to scavenge free radicals.

It is worth mentioning that, in a previous study, we have shown that the aqueous extract of *N. retusa* fruit exhibited a significant protective effect against penconazole-induced oxidative stress in the liver of adult rats (Chaâbane et al. 2015). Based on our previous and present findings, penconazole toxicity in rats seems to be more pronounced in the liver than in the kidney. Indeed, penconazole treatment increased the hepatic levels of MDA, H_2_O_2,_ AOPP, NPSH, MT, SOD, CAT and GPx by 103, 56, 181, 23, 17, 36, 52 and 52%, respectively. In line with this, tests on short-term toxicity have shown that the liver is the main target organ for this fungicide (EFSA 2008). As for the beneficial effect of *N. retusa* extract administration on penconazole toxicity, this plant extract showed to be more effective in reducing the elevation of MDA (−41%), H_2_O_2_ (−14%), AOPP (−44%), NPSH (−10%) and CAT (−22%) levels caused by penconazole in the liver of adult rats than in the renal tissue. While, it was found to be less successful in decreasing the rise of MT (−8%), SOD (−9%) and GPx (−13%) levels induced by penconazole in the hepatic tissue as compared to the present findings. So, the hepatoprotective potential of *N. retusa* extract appears to be more potent than its nephroprotective activity, especially in regards to its modulatory effects on lipid and protein oxidation.

The biochemical findings obtained in the current work were substantiated with histopathological observations. The renal tubules as well as the glomeruli were affected after penconazole treatment. In fact, kidney sections of penconazole-treated rats showed a renal tubular damage characterized by necrosis of the epithelial cells lining the tubules. This fungicide caused also an infiltration of leucocytes. These observations could be due to the accumulation of free radicals as a consequence of a lipid peroxidation increase in the renal tissue of penconazole-treated rats. Similar histopathological observations have been reported in kidney of rats treated with the metiram, another fungicide (Sakr et al. 2013). Moreover, kidney of rats exposed to penconazole showed glomeruli fragmentation and an enlarged Bowman’s space which matched with the previous findings of Shah and Iqbal (2010) in diazinon-treated rats.

Kidney histoarchitecture impairment, induced by penconazole, was remarkably reduced by the aqueous extract of *N. retusa* fruit confirming, consequently, the biochemical results.

Polyphenol constituents of *N. retusa* fruit aqueous extract might participate with their antioxidant activities in nephroprotection. In fact, the analysis of this extract by HPLC-HRMS is the first study which identified the phenolic compounds, including phenolic acids and flavonoids, whose major components were (3′-*O*-methyl-(-)-epicatechin 7-*O*-glucuronide, 4′-*O*-methyl-(-)-epicatechin 3′-*O*-glucuronide, epicatechin 3′-*O*-glucuronide, p-coumaric acid, cyanidin 3-*O*-rutinoside, 3-*O*-methylgallic acid, taxifoline and kaempferol 3-glucoside). They have been reported for their powerful antioxidant activity (Natsume et al. 2004; Luceri et al. 2007; Mulabagal et al. 2007; Gargouri et al. 2013; Topal et al. 2015; Wang et al. 2015). Furthermore, the nephroprotective effect of chlorogenic acid against cisplatin-induced kidney injury has been previously reported by Domitrović et al. (2014). In addition, Vijayaprakasha et al. (2013) have shown that kaempferol alleviates the toxicity induced by mercuric chloride in the kidney of rats. According to Zadernowski et al. (2005), hydroxycaffeic acid has been found to be the major phenolic acid in blackberries but information regarding its biological activities seems to be lacking. In general, polyphenols have been shown to attenuate renal dysfunction, to decrease lipid peroxidation and ROS, and to improve renal histoarchitecture (Wongmekiat et al. 2008). The renoprotective effect of phenolic compounds is thought to be essentially due to their wide range of biological actions, such as free radical scavenging, metal chelation and enzyme modulation capacities (Pietta et al. 1998). In particular, flavonoids represent an important class of phenolic compounds and have been reported to inhibit xenobiotic-induced nephrotoxicity in animal models (Devipriya & Shyamaladevim 1999). They exhibit a potent antioxidant effect, which is attributed to their free radical scavenging properties, their ability to reduce free radicals formation (Pietta 2000) and to stabilize membranes by decreasing their fluidity (Arora et al. 2000), which may inhibit the lipid peroxidation process. Furthermore, the mineral composition of *N. retusa* fruit could contribute to its antioxidant property. Trace elements such as Fe^2+^, Zn^2+ ^and Cu^2+ ^have been reported to play the key roles as cofactors for endogenous antioxidant enzymes (Powell 2000; Michalak 2006). The biological activities of these microelements are related to the presence of unpaired electrons allowing their participation in redox reactions.

Overall, our results suggested that the main mechanism underlying penconazole nephrotoxicity in rats involve the generation of ROS, resulting in the subsequent formation of oxidative stress in the renal tissue. According to the literature data, increased production of ROS can activate redox sensitive transcription factors, such as nuclear factor-κB (NF-κB), and signal transduction pathways, like mitogen-activated protein kinases (MAPKs). These events may lead to tissue damage and dysfunction by promoting several disorders such as necrosis, apoptosis, inflammation and fibrosis. Indeed, NF-κB is a ubiquitous transcription factor governing the expression of genes encoding a series of proinflammatory cytokines and chemokines. Its activation plays, therefore, a pivotal role in the pathogenesis of inflammation, tissue injury and renal disease (An et al. 2009). MAPKs represent a family of structurally related serine/threonine kinase enzymes, which include the extracellular-receptor kinases (ERK1/2), the c-Jun N-terminal kinases (JNK) and the p38 MAPKinases. The MAPKs pathway operates a crucial signalling pathway of cell death and inflammation in kidney (Xu et al. 2014). In this context, it has been demonstrated, in animal models of diabetic nephropathy, that ROS mediate the activation of various signalling pathways induced by high glucose, including MAPKs and NF-κB, and up-regulate genes and proteins involved in glomerular mesangial expansion and tubule-interstitial fibrosis (Lee et al. 2003). On the other hand, the nuclear factor erythroid-2-related factor (Nrf2), another transcription factor, is considered to be a main defence mechanism against oxidative stress in cells (Li et al. 2008). Nrf2 expression and its nuclear translocation have been involved in the induction of antioxidant defences and phase II detoxifying enzymes (Xu et al. 2014). In an experimental model of acute kidney injury in mice, Sahu et al. (2015) found that baicalein, a bioflavonoid, prevents cisplatin-induced renal injury by up-regulating Nrf2 expression and down-regulating the MAPKs and NF-κB pathways. Hence, to improve our understanding of the molecular mechanisms of penconazole-induced nephrotoxicity, it would be interesting to examine, in the future studies, the target pathways activated by ROS in renal cells exposed to this fungicide and the protective effects of *N. retusa* fruit extract.

## Conclusions

Our study provided a new knowledge regarding the phenolic composition of *N. retusa* fruit aqueous extract which showed to be a source of phenolic acids and flavonoids. Moreover, we demonstrated the beneficial contribution of this extract in mitigating penconazole-induced nephrotoxicity in adult rats. Therefore, *N. retusa* fruit aqueous extract could be used as a potential food supplement for the treatment of penconazole-induced kidney injury.

## References

[CIT0001] Abdelhalim MAK, Moussa SAA 2013 The biochemical changes in rats' blood serum levels exposed to different gamma radiation doses. Afr J Pharm Pharmacol. 7:785–792.

[CIT0002] Abd-Alrahman SH, Elhalwagy MEA, Kotb GA, Farid H, Farag AAG, Draz HM, Isa AM, Sabico S 2014 Exposure to difenoconazole, diclofop-methyl alone and combination alters oxidative stress and biochemical parameters in albino rats. Int J Clin Exp Med. 7:3637–3646. 25419412 PMC4238557

[CIT0003] Aebi H 1984 Catalase *in vitro*. Meth Enzymol. 105:121–126. 6727660 10.1016/s0076-6879(84)05016-3

[CIT0004] Ahmed NS, Mohamed AS, Abdel-Wahhab MA 2010 Chlorpyrifos induced oxidative stress and histological changes in retinas and kidney in rats: protective role of ascorbic acid and α tocopherol. Pestic Biochem Phys. 98:33–38.

[CIT0005] Alvarez-Lario B, Macarron-Vicente J 2010 Uric acid and evolution. Rheumatology. 49:2010–2015. 20627967 10.1093/rheumatology/keq204

[CIT0006] An WS, Kim HJ, Cho KH, Vaziri ND 2009 Omega-3 fatty acid supplementation attenuates oxidative stress, inflammation, and tubule-interstitial fibrosis in the remnant kidney. Am J Physiol Renal Physiol. 297:895–903. 10.1152/ajprenal.00217.2009 19656915

[CIT0007] Arora A, Byrem TM, Nair MG, Stasburg GM 2000 Modulation of liposomal membrane ﬂuidity by ﬂavonoids and isoﬂavonoids. Arch Biochem Biophys. 373:102–109. 10620328 10.1006/abbi.1999.1525

[CIT0008] Association of Official Analytical Chemists (AOAC) 2000 Official methods of analysis. 17th ed Gaithersburg, MD, USA: AOAC International.

[CIT0009] Badgujar PC, Pawar NN, Chandratre GA, Telang AG, Sharma AK 2014 Fipronil induced oxidative stress in kidney and brain of mice: protective effect of vitamin E and vitamin C. Pest Biochem Physiol. 118:10–18. 10.1016/j.pestbp.2014.10.013 25752424

[CIT0010] Banerjee BD, Seth V, Ahmed RS 2001 Pesticide-induced oxidative stress: perspectives and trends. Rev Environ Health. 16:1–40. 11354540 10.1515/reveh.2001.16.1.1

[CIT0011] Barnham KJ, Masters CL, Bush AI 2004 Neurodegenerative diseases and oxidative stress. Nat Rev Drug Discov. 3:205–214. 15031734 10.1038/nrd1330

[CIT0012] Beauchamp C, Fridovich I 1971 Superoxide dismutase: improved assays and an assay applicable to acrylamide gels. Anal Biochem. 44:276–287. 4943714 10.1016/0003-2697(71)90370-8

[CIT0013] Ben Amara I, Karray A, Hakim A, Ben Ali Y, Troudi A, Soudani N, Boudawara T, Zeghal KM, Zeghal N 2013 Dimethoate induces kidney dysfunction, disrupts membrane-bound ATPases and confers cytotoxicity through DNA damage. Protective effects of vitamin E and selenium. Biol Trace Elem Res. 156:230–242. 24114344 10.1007/s12011-013-9835-0

[CIT0014] Benvenuti S, Pellati F, Melegari M, Bertelli D 2004 Polyphenols, anthocyanins, ascorbic acid, and radical scavenging activity of *Rubus*, *Ribes*, and *Aronia*. J Food Sci. 69:164–169.

[CIT0015] Bhinder P, Chaudhry A 2013 Mutagenicity assessment of organophosphates using polymerase chain reaction-restriction fragment length polymorphism assay. Toxicol Int. 20:254–260. 24403735 10.4103/0971-6580.121678 PMC3877493

[CIT0016] Cabras P, Angioni A 2000 Pesticide residues in grapes, wine, and their processing products. J Agric Food Chem. 48:967–973. 10775335 10.1021/jf990727a

[CIT0017] Chaâbane M, Maktouf S, Sayari N, Zouari S, Zeghal N, Ellouze Ghorbel R 2014 Antioxidant and antimicrobial properties of the extracts from *Nitraria retusa* fruits and their applications to meat product preservation. Ind Crops Prod. 55:295–303.

[CIT0018] Chaâbane M, Soudani N, Benjeddou K, Turki M, Ayadi Makni F, Boudawara T, Zeghal N, Ellouze Ghorbel R 2015 The protective potential of *Nitraria retusa* on penconazole-induced hepatic injury in adult rats. Toxicol Environ Chem. 97:1253–1264.

[CIT0019] Chaâbane M, Tir M, Hamdi S, Boudawara O, Jamoussi K, Boudawara T, Ellouze Ghorbel R, Zeghal N, Soudani N 2016 Improvement of heart redox states contributes to the beneficial effects of selenium against penconazole-induced cardiotoxicity in adult rats. Biol Trace Elem Res. 169:261–270. 26150403 10.1007/s12011-015-0426-0

[CIT0020] Charrel M 1991 Urée et créatinine. In: Sémiologie biochimique (Urea and creatinin). Paris: Ellipses Publishers; p. 124–128.

[CIT0021] Council of European Communities 1986 Council instructions about the protection of living animals used in scientific investigations. OJEC (Jo 86/609/Cee). 358:1–18.

[CIT0022] Deneke SM 2000 Thiol-based antioxidants. Curr Top Cell Regul. 36:151–180. 10842751 10.1016/s0070-2137(01)80007-8

[CIT0023] Devipriya S, Shyamaladevim CS 1999 Protective effect of quercetin in cisplatin induced cell injury in the rat kidney. Indian J Pharmacol. 31:422–426.

[CIT0024] Domitrović R, Cvijanović O, Šušnić V, Katalinić N 2014 Renoprotective mechanisms of chlorogenic acid in cisplatin-induced kidney injury. Toxicology. 324:98–107. 25043994 10.1016/j.tox.2014.07.004

[CIT0025] Draper HH, Hadley M 1990 Malondialdehyde determination as index of lipid peroxidation. Meth Enzymol. 186:421–431. 2233309 10.1016/0076-6879(90)86135-i

[CIT0026] Durairaj AK, Mazumder UK, Gupta M, Ray SK 2007 Effects of methanolic extract of *Oxystelma esculentum* on diuresis and urinary electrolytes excretion in rats. Iran J Pharmacol Ther. 6:207–211.

[CIT0027] European Food Safety Authority (EFSA) 2008 Conclusion regarding the peer review of the pesticide risk assessment of the active substance penconazole. EFSA Sci Rep. 175:1–104. 10.2903/j.efsa.2008.147r PMC10193659 37213826

[CIT0028] El-Demerdash F, Dewer Y, El Mazoudy RH, Attia AA 2013 Kidney antioxidant status, biochemical parameters and histopathological changes induced by methomyl in CD-1 mice. Exp Toxicol Pathol. 65:897–901. 23375192 10.1016/j.etp.2013.01.002

[CIT0029] Ellman GL 1959 Tissue sulfhydryl groups. Arch Biochem Biophys. 82:70–77. 13650640 10.1016/0003-9861(59)90090-6

[CIT0030] El-Sharkawy EE, El-Nisr NA 2013 Testicular dysfunction induced by penconazole fungicide on male albino rats. Comp Clin Pathol. 22:475–480.

[CIT0031] Flohe L, Gunzler WA 1984 Assays of glutathione peroxidase. Methods Enzymol. 105:114–121. 6727659 10.1016/s0076-6879(84)05015-1

[CIT0032] Galal MK, Khalaf AAA, Ogaly HA, Ibrahim MA 2014 Vitamin E attenuates neurotoxicity induced by deltamethrin in rats. BMC Complement Altern Med. 14:458–465. 25439240 10.1186/1472-6882-14-458 PMC4265463

[CIT0033] Gargouri OD, Gargouri B, Trabelsi SK, Bouaziz M, Abdelhedi R 2013 Synthesis of 3-*O*-methylgallic acid a powerful antioxidant by electrochemical conversion of syringic acid. Biochim Biophys Acta. 1830:3643–3649. 23434437 10.1016/j.bbagen.2013.02.012

[CIT0034] Goetz AK, Ren HZ, Schmid JE, Blystone CR, Thillainadarajah I, Best DS, Nichols HP, Strader LF, Wolf DC, Narotsky MG, et al 2007 Disruption of testosterone homeostasis as a mode of action for the reproductive toxicity of triazole fungicides in the male rat. Toxicol Sci. 95:227–239. 17018648 10.1093/toxsci/kfl124

[CIT0035] Greim H, Saltmiras D, Mostert D, Strupp C 2015 Evaluation of carcinogenic potential of the herbicide glyphosate, drawing on tumor incidence data from fourteen chronic/carcinogenicity rodent studies. Crit Rev Toxicol. 45:185–208. 25716480 10.3109/10408444.2014.1003423 PMC4819582

[CIT0036] Hegazy AK, Al-Rowaily SL, Faisal M, Alatar AA, El-Bana MI, Assaeed AM 2013 Nutritive value and antioxidant activity of some edible wild fruits in the Middle East. J Med Plants Res. 15:938–946.

[CIT0037] Hou B, Wu L 2010 Safety impact and farmer awareness of pesticide residues. Food Agric Immunol. 21:191–200.

[CIT0038] Hou Y, Zeng Y, Li S, Qi L, Xu W, Wang H, Zhao X, Sun C 2014 Effect of quercetin against dichlorvos induced nephrotoxicity in rats. Exp Toxicol Pathol. 66:211–218. 24594122 10.1016/j.etp.2014.01.007

[CIT0039] Jollow DJ, Mitchell JR, Zampaglione N, Gillette JR 1974 Bromobenzene-induced liver necrosis. Protective role of glutathione and evidence for 3,4-bromobenzene oxide as the hepatotoxic metabolite. Pharmacology. 11:151–169. 4831804 10.1159/000136485

[CIT0040] Kalendar S, Kalendar Y, Durak D, Ogutcu A, Uzunhisarcikli M, Cevrimli BS, Yildirim M 2007 Methyl parathion induced nephrotoxicity in male rats and protective role of vitamins C and E. Pest Biochem Physiol. 88:213–218.

[CIT0041] Kayali R, Cakatay U, Akcay T, Altug T 2006 Effect of alpha-lipoic acid supplementation on markers of protein oxidation in post-mitotic tissues of ageing rat. Cell Biochem Funct. 24:79–85. 15532093 10.1002/cbf.1190

[CIT0042] Koubaa M, Mhemdi H, Vorobiev E 2015 Seed oil polyphenols: rapid and sensitive extraction method and high resolution-mass spectrometry identification. Anal Biochem. 476:91–93. 25747847 10.1016/j.ab.2015.02.025

[CIT0043] Le Floc’h E 1983 Contribution à une étude Ethnobotanique de la Flore Tunisienne 2nd ed Ministère de l’enseignement Supérieur et de la Recherche Scientifique; Imprimerie Officielle de la République Tunisienne, Tunis, p. 136–137.

[CIT0044] Lee HB, Yu MR, Yang Y, Jiang Z, Ha H 2003 Reactive oxygen species-regulated signaling pathways in diabetic nephropathy. J Am Soc Nephrol. 14:241–245. 10.1097/01.asn.0000077410.66390.0f 12874439

[CIT0045] Li W, Khor TO, Xu C, Shen G, Jeong WS, Yu S, Kong AN 2008 Activation of Nrf2-antioxidant signaling attenuates NF-κB-inflammatory response and elicits apoptosis. Biochem Pharmacol. 76:1485–1489. 18694732 10.1016/j.bcp.2008.07.017 PMC2610259

[CIT0046] Lowry OH, Rosebrugh NJ, Farr AL, Randall RJ 1951 Protein measurement with the Folin phenol reagent. J Biol Chem. 193:265–275. 14907713

[CIT0047] Luceri C, Giannini L, Lodovici M, Antonucci E, Abbate R, Masini E, Dolara P 2007 *p*-Coumaric acid, a common dietary phenol, inhibits platelet activity *in vitro* and *in vivo*. Br J Nutr. 97:458–468. 17313706 10.1017/S0007114507657882

[CIT0048] Michalak A 2006 Phenolic compounds and their antioxidant activity in plants growing under heavy metal stress. Pol J Environ Stud. 15:523–530.

[CIT0049] Mulabagal V, van Nocker S, Dewitt DL, Nair MG 2007 Cultivars of apple fruits that are not marketed with potential for anthocyanin production. J Agric Food Chem. 55:8165–8169. 17822290 10.1021/jf0718300

[CIT0050] Natsume M, Osakabe N, Yasuda A, Baba S, Okunaga T, Kondo K, Osawa T, Terao J 2004 *In vitro* antioxidative activity of (-)-epicatechin glucuronide metabolites present in human and rat plasma. Free Radic Res. 38:1341–1348. 15763958 10.1080/10715760400022087

[CIT0051] Nayeem M, Quadri MFA 2015 Evaluation of diuretic activity of *Boswellia serrata* leaf extracts in albino mice. Int J Pharm Pharm Sci. 7:502–505.

[CIT0052] Nelson DL, Cox MM 2004 Lehninger Principles of Biochemistry. Sao Paulo: Sarvier; p. 1304.

[CIT0053] Ogutcu A, Suludere Z, Kalender Y 2008 Dichlorvos-induced hepatotoxicity in rats and the protective effects of vitamins C and E. Environ Toxicol Pharmacol. 26:355–361. 21791388 10.1016/j.etap.2008.07.005

[CIT0054] Ou P, Wolff SP 1996 A discontinuous method for catalase determination at near physiological concentrations of H_2_O_2_ and its application to the study of H_2_O_2_ fluxes within cells. J Biochem Biophys Methods. 31:59–67. 8926339 10.1016/0165-022x(95)00039-t

[CIT0055] Perry G, Nunomura A, Siedlak SL, Harris PLR, Xiongwei Z, Castellani RJ, Aliev G, Smith MA 2001 Oxidant and antioxidant responses in Alzheimer disease. Rec Res Dev Biophys Biochem. 1:35–41.

[CIT0056] Petrovic S, Ozretic B, Krajnovic-Ozretic M, Bobinac D 2001 Lysosomal membrane stability and metallothioneins in digestive gland of mussels (*Mytilus galloprovincialis* Lam.) as biomarkers in a field study. Mar Pollut Bull. 42:1373–1378. 11827125 10.1016/s0025-326x(01)00167-9

[CIT0057] Pietta P, Simonetti P, Gordana C, Brusamolino A, Morazzoni P, Bombardelli E 1998 Relationship between rate and extent of catechin absorption and plasma antioxidant status. Biochem Mol Biol Int. 46:895–903. 9861443 10.1080/15216549800204442

[CIT0058] Pietta PG 2000 Flavonoids as antioxidants. J Nat Prod. 63:1035–1042. 10924197 10.1021/np9904509

[CIT0059] Poovala VS, Vijaya KK, Tachikawa H, Salahudeen AK 1998 Role of oxidant stress and antioxidant protection in acephate-induced renal tubular cytotoxicity. Toxicol Sci. 46:403–409. 10048144 10.1006/toxs.1998.2559

[CIT0060] Pottier-Alapetite G 1979. Flowers of Tunisia: Angiosperms, dicotyledons, apetals, dialypetals Tunisia. Tunisia: Ministry of Higher Education and Scientific Research and the Ministry of Agriculture; p. 456.

[CIT0061] Powell SR 2000 The antioxidant properties of zinc. J Nutr. 130:1447–1454. 10.1093/jn/130.5.1447S 10801958

[CIT0062] Reznick AZ, Packer L 1994 Oxidative damage to proteins: spectrophotometric method for carbonyl Methods Enzymol. New York: Academic Press; p. 357–359. 10.1016/s0076-6879(94)33041-7 8015470

[CIT0063] Rice-Evans CA, Miller NJ, Bolwell PG, Bramlev PM, Pridham JB 1995 The relative antioxidant activities of plant-derived polyphenolic flavonoids. Free Radic Res. 22:375–383. 7633567 10.3109/10715769509145649

[CIT0064] Rodrigo R, Rivera G 2002 Renal damage mediated by oxidative stress: a hypothesis of protective effects of red wine. Free Radic Biol Med. 33:409–422. 12126763 10.1016/s0891-5849(02)00908-5

[CIT0065] Sahu BD, Kumar JM, Sistla R 2015 Baicalein, a bioflavonoid, prevents cisplatin-induced acute kidney injury by up-regulating antioxidant defenses and down-regulating the MAPKs and NF-κB pathways. PLOS One. 10:e0134139. 26222683 10.1371/journal.pone.0134139 PMC4519041

[CIT0066] Sakr S, EL-Kenawy A, EL-Sahra D 2013 Metiram-induced nephrotoxicity in albino mice: effect of licorice aqueous extract. Environ Toxicol. 28:372–379. 21544926 10.1002/tox.20728

[CIT0067] Sakr SA 2007 Ameliorative effect of ginger (*Zingiber officinale*) on mancozeb fungicide induced liver injury in albino rats. Aust J Basic Appl Sci. 1:650–656.

[CIT0068] Salahudeen AK 1995 Role of lipid peroxidation in H_2_O_2_-induced renal epithelial (LLC-PK1) cell injury. Am J Physiol. 268:30–38. 10.1152/ajprenal.1995.268.1.F30 7840245

[CIT0069] Sengupta T, Chattopadhay D, Ghosh N, Das M, Chatterjee GC 1990 Effect of chromium administration on glutathione cycle of rat intestinal epithelial cells. Indian J Exp Biol. 28:1132–1135. 2099328

[CIT0070] Shah MD, Iqbal M 2010 Diazinon-induced oxidative stress and renal dysfunction in rats. Food Chem Toxicol. 48:3345–3353. 20828599 10.1016/j.fct.2010.09.003

[CIT0071] Shaltout KH, Sheded MG, El-Kady HF, Al-Sodany YM 2003 Phytosociology and size structure of *Nitraria retusa* along the Egyptian Red Sea coast. J Arid Environ. 53:331–345.

[CIT0072] Takaori H 1993. Thiophanate-methyl combined chronic toxicity/oncogenicity study in rats. Unpublished report no.RD-9327 from Nisso Institute for Life Sciences, Kanagawa, Japan. Submitted to WHO by Nippon Soda Co. Ltd, Tokyo, Japan.

[CIT0073] Ting CM, Lee YM, Wong CKC, Wong AS, Lung HL, Lung ML, Lo KW, Wong RN, Mak NK 2010 2-Methoxyestradiol induces endoreduplication through the induction of mitochondrial oxidative stress and the activation of MAPK signaling pathways. Biochem Pharmacol. 79:825–841. 19883629 10.1016/j.bcp.2009.10.018

[CIT0074] Topal F, Nar M, Gocer H, Kalin P, Kocyigit UM, Gülçin I, Alwasel SH 2015 Antioxidant activity of taxifolin: an activity–structure relationship. J Enzyme Inhib Med Chem. 6:1–10. 10.3109/14756366.2015.1057723 26147349

[CIT0075] Viarengo A, Ponzano E, Dondero F, Fabbri R 1997 A simple spectrophotometric method for metallothionein evaluation in marine organisms: an application to Mediterranean and Antarctic molluscs. Mar Environ Res. 44:69–84.

[CIT0076] Vijayaprakasha S, Langeswaranb K, Kumar SG, Revathya R, Balasubramaniana MP 2013 Nephro-protective significance of kaempferol on mercuric chloride induced toxicity in Wistar albino rats. Biomed Aging Pathol. 3:119–124.

[CIT0077] Wang Y, Tang C, Zhang H 2015 Hepatoprotective effects of kaempferol 3-*O*-rutinoside and kaempferol 3-*O*-glucoside from *Carthamus tinctorius L.* on CCl_4_-induced oxidative liver injury in mice. J Food Drug Anal. 23:310–317. 28911387 10.1016/j.jfda.2014.10.002 PMC9351762

[CIT0078] Wongmekiat O, Leelarugrayub N, Thamprasert K 2008 Beneficial effect of shallot (*Allium ascalonicum* L.) extract on cyclosporine nephrotoxicity in rats. Food Chem Toxicol. 46:1844–1850. 18308444 10.1016/j.fct.2008.01.029

[CIT0079] Xavier R, Rekha K, Bairy KL 2004 Health perspective of pesticide exposure and dietary management. Malays J Nutr. 10:39–51. 22691747

[CIT0080] Xu J, Wang H, Ding K, Zhang L, Wang C, Li T, Wie W, Lu X 2014 Luteolin provides neuroprotection in models of traumatic brain injury via the Nrf2-ARE pathway. Free Radic Biol Med. 71:186–195. 24642087 10.1016/j.freeradbiomed.2014.03.009

[CIT0081] Xu W, Shao X, Tian L, Gu L, Zhang M, Wang Q, Wu B, Wang L, Yao J, Xu X, et al 2014 Astragaloside IV ameliorates renal fibrosis via the inhibition of mitogen-activated protein kinases and antiapoptosis *in vivo* and *in vitro*. J Pharmacol Exp Ther. 350:552–562. 24951279 10.1124/jpet.114.214205

[CIT0082] Yuliana ND, Khatib A, Link-Struensee AM, Ijzerman AP, Rungkat-Zakaria F, Choi YH, Verpoorte R 2009 Adenosine A1 receptor binding activity of methoxy flavonoids from *Orthosiphon stamineus*. Planta Med. 75:132–136. 19137497 10.1055/s-0028-1088379

[CIT0083] Zadernowski R, Naczk M, Nesterowicz J 2005 Phenolic acid profiles in some small berries. J Agric Food Chem. 53:2118–2124. 15769144 10.1021/jf040411p

[CIT0084] Zarn JA, Bruschweiler BJ, Schlatter JR 2003 Azole fungicides affect mammalian steroidogenesis by inhibiting sterol 14 alpha-demethylase and aromatase. Environ Health Perspect. 111:255–261. 12611652 10.1289/ehp.5785 PMC1241380

[CIT0085] Zhang X, Yang F, Zhang X, Xu Y, Liao T, Song S, Wang H 2008 Induction of hepatic enzymes and oxidative stress in Chinese rare minnow (*Gobiocypris rarus*) exposed to waterborne hexabromocyclododecane (HBCDD). Aquat Toxicol. 86:4–11. 18022707 10.1016/j.aquatox.2007.07.002

[CIT0086] Zou W, Yan M, Xu W, Huo H, Sun L, Zheng Z 2001 Cobalt chloride induces PC12 cells apoptosis through reactive oxygen species and accompanied by AP-1 activation. J Neurosci Res. 64:646–653. 11398189 10.1002/jnr.1118

